# The Impact of Genotyping-by-Sequencing Pipelines on SNP Discovery and Identification of Markers Associated with Verticillium Wilt Resistance in Autotetraploid Alfalfa (*Medicago sativa* L.)

**DOI:** 10.3389/fpls.2017.00089

**Published:** 2017-02-07

**Authors:** Long-Xi Yu, Ping Zheng, Suresh Bhamidimarri, Xiang-Ping Liu, Dorie Main

**Affiliations:** ^1^Plant Germplasm Introduction and Testing Research, United States Department of Agriculture-Agricultural Research Service, ProsserWA, USA; ^2^Department of Horticulture, Washington State University, PullmanWA, USA; ^3^S&W Seed Company, ArlingtonWI, USA

**Keywords:** GWAS, GBS bioinformatics, Verticillium wilt, alfalfa

## Abstract

Verticillium wilt (VW) of alfalfa is a soilborne disease causing severe yield loss in alfalfa. To identify molecular markers associated with VW resistance, we used an integrated framework of genome-wide association study (GWAS) with high-throughput genotyping by sequencing (GBS) to identify loci associated with VW resistance in an F1 full-sib alfalfa population. Phenotyping was performed using manual inoculation of the pathogen to cloned plants of each individual and disease severity was scored using a standard scale. Genotyping was done by GBS, followed by genotype calling using three bioinformatics pipelines including the TASSEL-GBS pipeline (TASSEL), the Universal Network Enabled Analysis Kit (UNEAK), and the haplotype-based FreeBayes pipeline (FreeBayes). The resulting numbers of SNPs, marker density, minor allele frequency (MAF) and heterozygosity were compared among the pipelines. The TASSEL pipeline generated more markers with the highest density and MAF, whereas the highest heterozygosity was obtained by the UNEAK pipeline. The FreeBayes pipeline generated tetraploid genotypes, with the least number of markers. SNP markers generated from each pipeline were used independently for marker-trait association. Markers significantly associated with VW resistance identified by each pipeline were compared. Similar marker loci were found on chromosomes 5, 6, and 7, whereas different loci on chromosome 1, 2, 3, and 4 were identified by different pipelines. Most significant markers were located on chromosome 6 and they were identified by all three pipelines. Of those identified, several loci were linked to known genes whose functions are involved in the plants’ resistance to pathogens. Further investigation on these loci and their linked genes would provide insight into understanding molecular mechanisms of VW resistance in alfalfa. Functional markers closely linked to the resistance loci would be useful for MAS to improve alfalfa cultivars with enhanced resistance to the disease.

## Introduction

Verticillium wilt (VW) is a soil-borne disease caused by the fungal pathogen *Verticillium alfalfae* (Renormalized by Inderbitzin et al., 2011) in alfalfa (*Medicago sativa* L.). It causes severe forage yield loss in US and Canada (Graham et al., 1977; Atkinson, 1981; Grau et al., 1981; Gray and Roth, 1982; Gordon et al., 1989). The disease symptoms start with leaf tip chlorosis, leaf desiccation and abscission, and then, as disease progresses, infected plants eventually wilt and die (Leath and Pennypacker, 1990). Methods such as eradication of broadleaf weed hosts and crop rotation may be used for controlling the disease (Leath and Pennypacker, 1990). However, due to other dispersal factors including insects, manure, wind, and water, these control strategies are often ineffective. For this reason, the preferred method of control of the disease is the use of resistant varieties (Peaden et al., 1985; Leath and Pennypacker, 1990). It has been suggested that cultivars require at least 60% resistant plants for reasonable protection against the disease (Grau, 1991).

Efforts toward the goal of developing alfalfa cultivars resistant to VW have been made using a traditional breeding strategy (Delwiche, 1991). However, the traditional strategy for selecting resistant cultivars is based on plant’s reaction to the disease (Delwiche, 1991), and the effect of environmental factors on disease expression leads to difficulty in accurately screening alfalfa for resistance to VW. Development of high-throughput diagnostic markers linked to the plant resistance gene(s) would enable more robust breeding strategies, as they can be used in marker-assisted selection of resistant varieties in a timely manner. Single-nucleotide polymorphism (SNP) markers have become the technology of choice for most organisms because of their high frequency, wide distribution in genomes, and ready adaptation to highly multiplex detection systems.

Quantitative traits such as biotic and abiotic stress resistance are most likely under the control of multiple genes and interact with environmental factors. Identification of resistance loci that contribute to variation in such complex traits is a primary challenge in plant breeding and population genetics. An integrated framework that merges a QTL mapping approach called a “genome-wide association study (GWAS)” with high-throughput genome sequencing methodologies called “genotyping by sequencing (GBS)” provide a statistical basis for analyzing marker-trait association using linkage disequilibrium, and help to map traits quickly, efficiently, and in a relatively inexpensive manner. GBS generates large raw datasets of sequence reads and requires bioinformatics pipelines to analyze and interpret the GBS datasets. Current GBS pipelines such as TASSEL-GBS (TASSEL) (Glaubitz et al., 2014) and the Universal Network Enabled Analysis Kit (UNEAK) (Lu et al., 2013) have been applied for SNP discovery. The TASSEL pipeline was initially developed for diploid species but has also been used successfully in polypoid species such as wheat and barley (Poland et al., 2012). UNEAK is a non-reference, network-based pipeline. Although it has been used for genotype calling in polypoid species such as switch grass (Lu et al., 2013) and alfalfa (Li et al., 2014; Annicchiarico et al., 2015, Annicchiarico et al., 2016), it has been a challenge for GBS genotype calling in autotetraploid species such as alfalfa and potato due to their outcrossing and high heterozygosity. Recently, the software FreeBayes has been developed (Garrison and Marth, 2012) and used for GBS genotype calling in autotetraploid potato (Uitdewilligen et al., 2013) and alfalfa (Zhang et al., 2015). As FreeBayes is a haplotype-based pipeline, variants including single and multiple nucleotide polymorphisms (SNPs and MNPs, respectively), allelic series of tri-SNPs and tetra-SNPs, MNPs, and indels with a variable number of nucleotides, and nulliplex (aaaa), simplex (aaab), duplex (aabb), triplex (abbb) and quadruplex (bbbb) genotypes can be identified for the tetraploid samples. In a previous report, we used FreeBayes in combination with other software such as Stacks (Catchen et al., 2011) and GATK (McKenna et al., 2010) for GBS SNP discovery and identified loci associated with VW resistance in a breeding population of alfalfa developed by Forage Genetics International (Yu et al., 2016).

Cultivated alfalfa is an allogamous autotetraploid (2n = 4x = 32) with a basic chromosome number of eight and a genome size of 800–1000 Mbp (Blondon et al., 1994). Alfalfa plants are highly heterozygous and exhibit strong inbreeding depression. Selfing can cause either self-sterility or lethal allelic combinations. F2 populations may suffer from a genetic bias induced by the death of some genotypes (Julier et al., 2003). It is a considerable challenge to genotype individuals with such a complicated genome. To address whether different GBS pipelines affect SNP discovery in alfalfa, in the present study, we carried out GBS in an F1 alfalfa population developed by S & W Seeds for selecting for VW resistance. We compared three GBS pipelines, including the TASSEL, UNEAK and FreeBayes pipelines for genotype calling using the same GBS data set. The resulting marker data set generated by each pipeline was used for marker-trait association using linkage disequilibrium. Markers associated with VW were identified and compared among different pipelines for the effect of pipelines on SNP discovery and marker identification.

## Materials and Methods

### Plant Materials and Phenotyping

An alfalfa population containing 188 F_1_ progeny was developed from a cross between the parental plants of 55V50-58 (resistant) × 55V50-118 (susceptible) at S & W Seeds. The two parents and the F_1_ progeny were propagated by stem cuttings. Three clones from each individual were evaluated for resistance to VW by the standard assay of the North American Alfalfa Improvement Conference^1^ as described previously by Zhang et al. (2014).

The disease severity scores of three replicated clones were obtained for each individual and analyzed using Levene’s and *t*-tests to evaluate the equality of variance and means (**Table 1**). Based on the assumption of equal variance, a test for normality was performed on the obtained disease severity scores of the population with the Kolmogorov–Smirnov tests (**Table 1**) using the SPSS software^2^. Least square means were estimated from 3 replicated disease severity scores using the SAS PROC mixed (SAS Institute Inc. 2011, SAS Online Doc 9.3, Cary, NC, USA) and used for association mapping.

**Table 1:** The Levene’s and *t*-tests, and the normal distribution of the Shapiro–Wilk and Kolmogorov–Smirnov tests.

		Levene’s and *t*-tests					Normal distribution		
Variance	*n*	Mean	Standard error	Equality of variance	Equality of means	Method	Parameter estimate	*p*-value	Significant
VW	188	2.75	0.08	*P* = 0.513	*P* = 0.08	Shapiro–Wilk	*W* = 0.899	5.66E-10	^∗∗∗∗^
						Kolmogorov	*D* = 0.209	1.41E-07	^∗∗∗^

### Genotyping by Sequencing

High molecular weight DNA was extracted from leaves of the original plants used to make clones using the Qiagen DNeasy 96 Plant kit, following the manufacturer’s protocol (Qiagen, CA). Genotyping-by-sequencing was carried out as described by Elshire et al. (2011). Briefly, DNA samples from individuals of the association panel were digested by ApekI restriction enzyme. GBS libraries were prepared by ligating the digested DNA to unique nucleotide adapters (barcodes) in 96-plex, followed by PCR amplification. Sequencing was performed on the Illumina HiSeq2000 instrument using two lanes.

**FIGURE 1:**
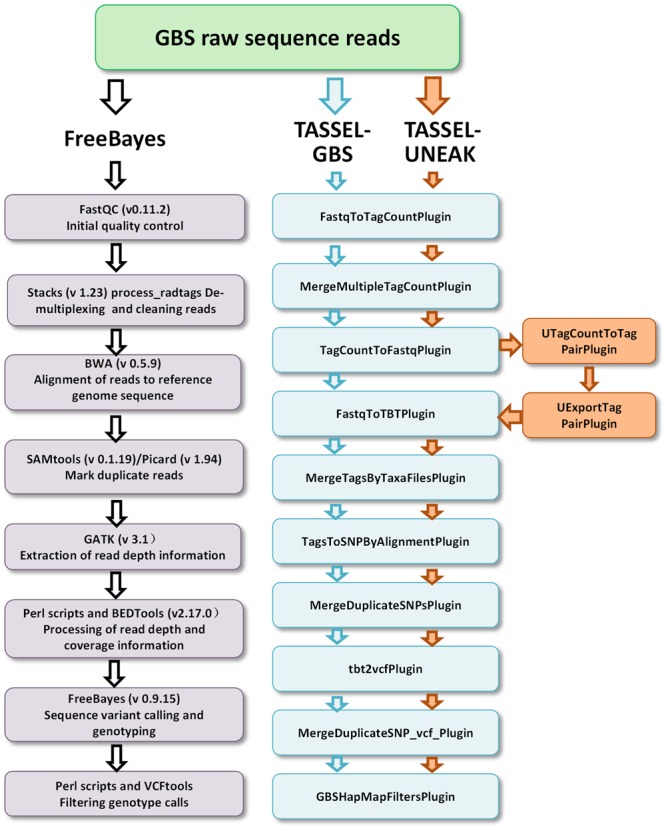
**Three GBS pipelines including FreeBayes **(left)**, TASSEL-GBS **(medium)** and TASSEL-UNEAK **(medium and right)** used for genotype calling.** Bioinformatics steps are presented in each pipeline. Note that the TASSEL-GBS and UNEANK share most of steps in their pipelines except additional steps are used in the UNEAK pipeline **(right)**.

Three GBS pipelines, the FreeBayes pipeline, the TASSEL-GBS (TASSEL) pipeline, and the Universal Network-Enabled Analysis Kit (UNEAK) pipeline, were used for SNP/variant discovery. The FreeBayes and TASSEL pipelines used the reference genomic sequences of *M. truncatula* (Mt4.0, v1), while no reference sequence was used in the UNEAK pipeline. The working flows of the pipelines are outlined in **Figure 1**. For the FreeBayes pipeline (**Figure 1**, left panel), GBS raw sequence reads were quality checked using FastQC (v0.11.2), followed by demultiplexing with STACKS (v 1.23) (Catchen et al., 2011). Clean reads were then aligned to the reference genome sequence of *M. truncatula* using the Burrows–Wheeler Aligner (BWA) (Li and Durbin, 2009). SAMtools (version 0.1.19) (Durbin et al., 2009) and Picard (version 1.94^3^) were then used to mark duplicate reads and estimate the average insert size of the single-end reads. The read-depth and coverage data was processed with the Genome Analysis Toolkit (GATK) (McKenna et al., 2010), and in-house Perl scripts and BEDTools (Quinlan and Hall, 2010). Sequence variants were called using FreeBayes (Version 0.9.15) (Garrison and Marth, 2012) after removing duplicate reads. Consequently, haplotype-based variants including SNPs and MNPS (single and multiple nucleotide polymorphisms, respectively), allelic series of tri-SNPs and tetra-SNPs, MNPs, and indels with a variable number of nucleotides were identified. Default parameters were used for estimating pairwise diversity. A minimal read depth of 15X at a specific variant position was used for identifying an alternative allele as a variant. The QUAL was used for estimating the phred-scaled probability and sites with a QUAL value less than 20 were removed. Using these criteria, an estimated error rate of less than 0.01 was obtained. The R-package “pegas”^4^ and custom Perl scripts were used for calculating the heterozygosity of variants.

For the TASSEL-GBS pipeline, a TASSEL_Plugin interface with default parameters^5^ was used to call SNPs (**Figure 1**, intermediate). Tag counts were generated from FastQ files with the FastqToTagCountPlugin. The tag counts were merged with the MergeMultipleTagCountPlugin, the minimum number of five times presence for a tag was required for output. Tags were then aligned to the reference genome of *M. truncatula* using BWA. Tags were converted to FastQ with the TagCountToFastqPlugin. Counts of tags per individual (taxa) were generated with the FastqToTBTPlugin. Counts of tags per individual were merged with the MergeTagsByTaxaFilesPlugin. SNPs were called using the TagsToSNPByAlignmentPlugin. Duplicate sites were merged with the MergeDuplicateSNPsPlugin and then with the MergeDuplicateSNP_vcf_Plugin followed by the tbt2vcfPlugin. Finally, a filtered HapMap containing GBS SNPs was formatted with the GBSHapMapFiltersPlugin (**Figure 1**, medium panel). The minimums of site coverage of 0.8, taxa coverage of 0.1 and MAF of 0.01 were applied in the filter.

For the UNEAK pipeline, a network approach was used for discovering SNPs without a reference genome as described by Lu et al. (2013). It was implemented in the TASSEL-GBS software program with two additional steps, UTagCountToTagPairPlugin and then UExportTagPairPlugin. They were added to the TASSEL pipeline between the TagcountToFastqPlugin and the FastqToTBTPlugin steps (**Figure 1**, right panel). The same parameters used in TASSEL-GBS were used for UNEAK except that an error tolerance rate of 0.03 was used in the network filter and a distance of 1,000 was used for padding tag pairs.

The GBS data have been submitted to the NCBI Sequence Read Archive with the BioProject ID: PRJNA343543.

### Marker-Trait Association

To estimate effects of genotype and its interaction with environment, we used least square means of replicated disease scores to evaluate associations. A mixed linear model (MLM) was used for analyzing marker-trait association using TASSEL (Bradbury et al., 2007). Each set of marker data generated by the TASSEL, UNEAK and FreeBayes pipelines were further filtered using 5% MAF and 25% missing values before association mapping. Each filtered marker data and the same set of phenotypic data were used for association mapping. To correct for population structure, Kinship and principal component analysis were carried out for generating K and Q matrices, respectively. The K and Q matrices were used as covariance and integrated into the MLM for controlling for population structure. A false discovery rate (FDR) of 0.05 was used as multiple-test correction for significantly associated markers (Benjamini and Hochberg, 1995).

### BLAST Search for Putative Candidate Genes

Flanking sequences of significant markers (Supplementary Table S4) were used as queries for BLAST search in the DNA database of the National Centre for Biotechnology Information (NCBI^6^) and Phytozome against the *Medicago truncatula* genome sequence, Mt4.0 v1^7^. Known genes linked to the significant loci were assigned as putative candidates based on the annotation of gene functions.

## Results

### Analysis of Phenotypic Data

For phenotyping VW resistance, the foliar symptoms of individual plants were evaluated after pathogen inoculation in the greenhouse and scored for VW resistance. Resistance scores of 3 replications were analyzed for the 190 individuals (570 plants total). The *P*-value for equality of variances (Levene’s Test) was less than 0.001 (**Table 1**). The *t*-test for equality was significant (*p* < 0.05). A normal distribution with a mean of 2.75 and a standard error of 0.08 was observed (Shapiro–Wilk test, **Table 1**).

### Genotype Calling by Different Pipelines

**FIGURE 2:**
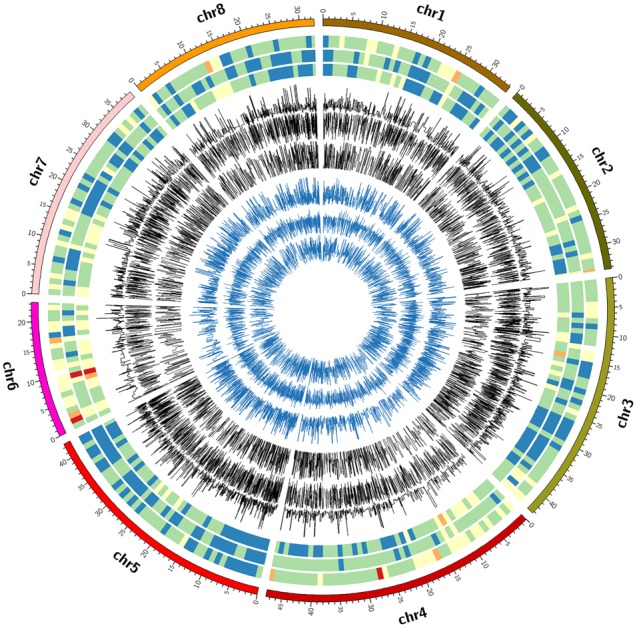
**Circle plot of chromosome location, marker density, minor allele frequency and heterozygosity of valid SNPs by three GBS pipelines.** From outside to inside: circle 1 = chromosome location, circles 2, 3, and 4 = marker density, circle 5, 6, and 7 = minor allele frequency, circle 8, 9, and 10 = heterozygosity by FreeBayes, TASSEL and UNEAK, respectively. The colors for marker density were Red < brown < yellow < green < gray < blue.

**FIGURE 3:**
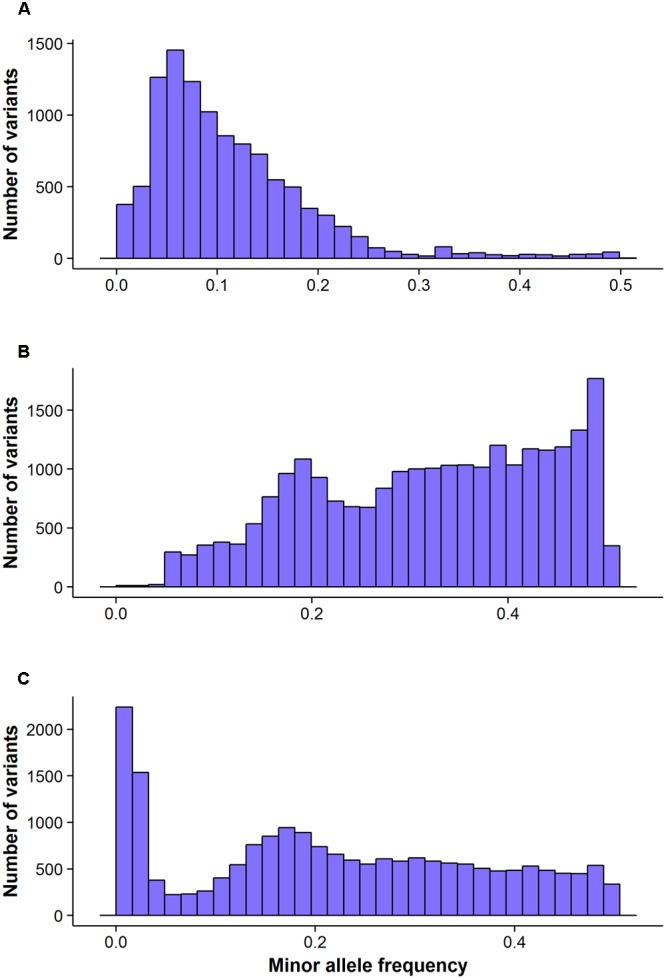
**The distribution of minor allele frequency of valid variants calls by different GBS pipelines:** FreeBayes **(A)**, TASSEL **(B)** and UNEAK **(C)**.

**FIGURE 4:**
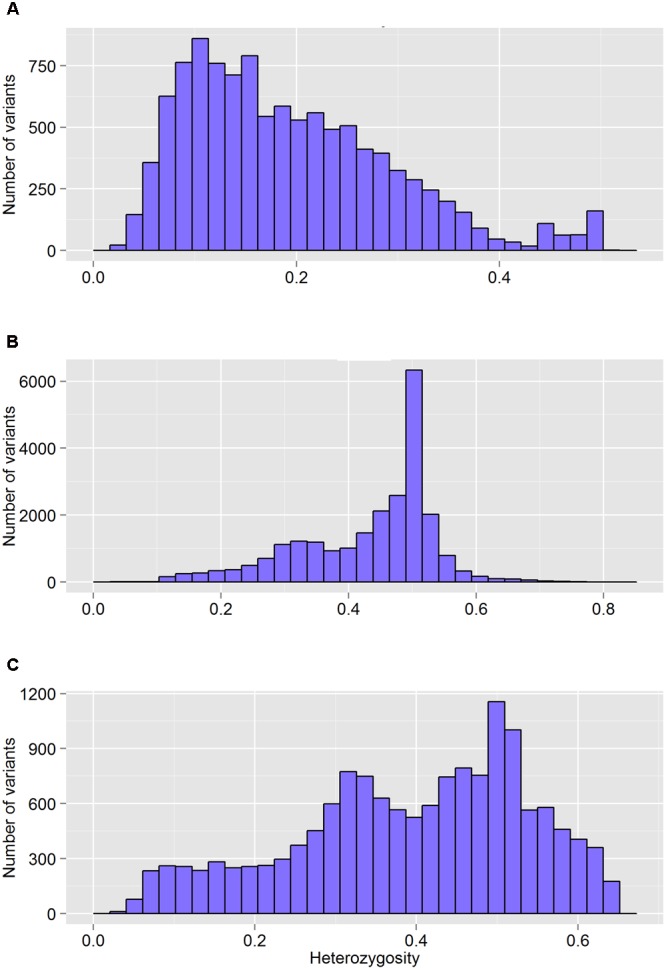
**The distribution of heterozygosity of valid variants after genotyping by different GBS pipelines: FreeBayes (A)**, TASSEL **(B)**, and UNEAK **(C)**.

The FreeBayes pipeline generated 10,403 valid variants after filtering (**Figure 2**, Supplementary Table S1). The site depth was 1.3 with 0.28 of missing value (**Table 2**). The average minor allele frequency (MAF) was 0.11 (**Figure 3A**). The average heterozygosity was 0.19 (**Figure 4A**) with 0.5% SNPs having heterozygosity ≥ 0.5 (**Table 2**).

**Table 2:** Total number, density, minor allele frequency and heterozygosity of the filtered variants generated by different GBS pipelines.

Pipeline	Number of variants	Site depth	Site missing	MAF	Hetero-zygosity	% heter ≥ 0.5
FreeBayes	10,403	1.3	0.28	0.11	0.19	0.5
TASSEL	24,176	8.8	0.31	0.32	0.43	35
UNEAK	14,415	3.5	0.5	0.22	0.39	28

*MAF, minor allele frequency; % heter ≥ 0.5, percentage of heterozygosity equal or more than 0.5.*

The TASSEL pipeline generated 24,176 valid variants (**Figure 2**, Supplementary Table S2). The site depth was 8.8 with 0.31 missing value (**Table 2**). The averages of MAF and heterozygosity were 0.32 (**Figure 3B**) and 0.43 (**Figure 4B**), respectively, and 35% SNPs with heterozygosity ≥ 0.5 (**Table 2**).

The UNEAK pipeline generated 14,415 valid variants with site depth of 3.5 and 0.5 missing value (Supplementary Table S3, **Figure 2**). The average of MAF was 0.22 (**Figure 3C**). The average heterozygosity was 0.39 (**Figure 4C**), and 28% SNPs with heterozygosity ≥ 0.5 (**Table 2**).

### Marker-Trait Association

**FIGURE 5:**
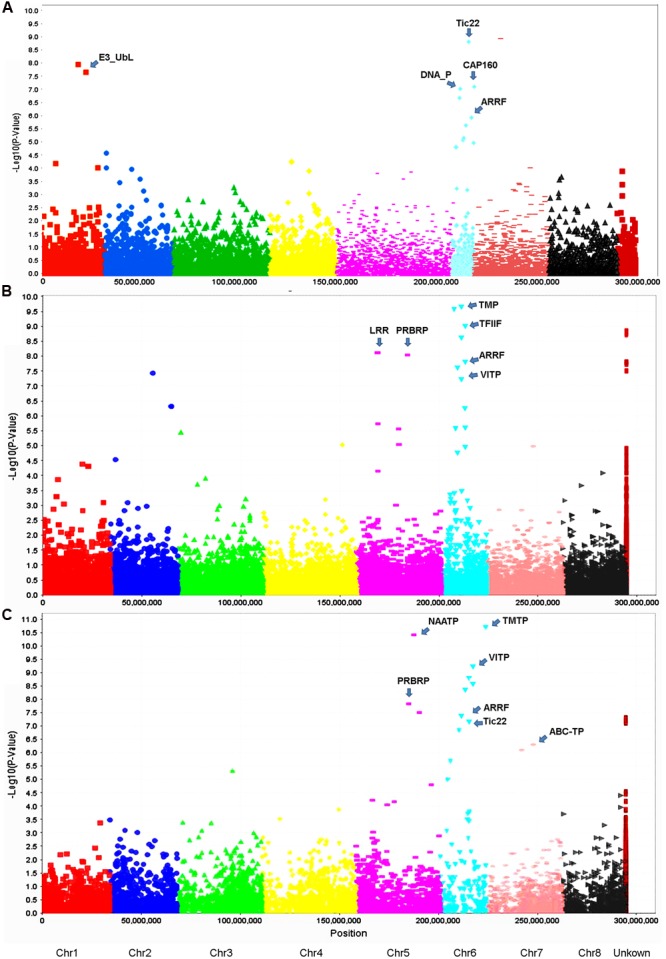
**Manhattan and quantile–quantile plots resulting from GWAS using the FreeBayes (A)**, TASSEL-GBS **(B)** and UNEAK **(C)** pipelines in the S & W Seeds population. A false discovery rate of 0.05 was used for significant cutoff (dot lines). Significant markers (above the cutoff lines) associated with VW resistance are listed in **Tables 3–5**, respectively. The chromosome positions were physical positions (not genetic positions) based on the alignment of alfalfa sequence tags to the reference genome of *M. truncatula* sequence (Version 3.5). “U” represents unknown chromosome. The abbreviations are putative candidate genes linked to the respective significant marker loci and the arrows indicate their genetic positions in the reference genome. ABC-TP, ABC transporter; ARRF, ankyrin repeat RF-like protein; CAP160, CAP160 repeat protein; DNA_P, DNA primase; E3_UbL, E3 ubiquitin protein ligase XBOS34; LRR, LRR receptor-like kinase family protein; NAATP, neutral amino acid transporter; PRBRP, pumilio-family RNA-binding protein; TFIIF, transcription initiation factor IIF beta subunit; TMP, transmembrane protein; TMTP, transmembrane amino acid transporter family protein; Tic22, Tic22 family protein; VITP, vacuolar iron transporter-like protein.

Using the criteria mentioned in Section “Materials and Methods,” we have identified 13 GBS markers from the FreeBayes pipeline significantly associated with VW resistance (**Table 3**, **Figure 5A**). BLAST search of the flanking sequences of the markers against *M. truncatula* pseudomolecule Mt4.0 v1^8^ revealed that they were located on three chromosomes (**Table 3**). Among them, eight markers were located on chromosome 6, four on chromosome 1 and one on chromosome 7. The *p*-values ranged from 7.0E-6 to 8.0E-10 with *R*^2^ values of 0.12 – 0.20. The most significant marker was S7_17986403 with *p* = 8.0E-10 and *R*^2^ of 0.20 (**Table 3**).

**Table 3:** Significant markers associated with VW by the FreeBayes pipeline.

Trait	Marker	Allele	Chr	*P*-value	*R*^2^	Candidate
VW	S1_22968515	G/T	1	1.41E-08	0.18	
VW	AC146807-58_93588	C/A	1^∗^	7.48E-08	0.15	E3_UbL
VW	AC146807-58_93595	G/C	1^∗^	7.48E-08	0.15	E3_UbL
VW	AC146807-58_93603	TTATGG/TG	1^∗^	9.11E-08	0.14	E3_UbL
VW	S6_10615509	G/T	6	1.40E-07	0.14	DNA_P
VW	S6_10615526	C/T	6	6.11E-08	0.15	DNA_P
VW	S6_13861302	C/T	6	5.57E-06	0.12	
VW	Contig_100652_1157	T/A	6^∗^	1.56E-06	0.15	ARRF
VW	Contig_59661_3280	T/G	6^∗^	9.94E-10	0.18	Tic22
VW	Contig_73909_2401	A/T	6^∗^	7.69E-07	0.16	CAP160
VW	Contig_73909_2417	T/C	6^∗^	7.03E-06	0.14	CAP160
VW	Contig_73909_2437	C/T	6^∗^	5.14E-08	0.18	CAP160
VW	S7_17986403	T/G	7	8.05E-10	0.20	

*“Chr,” chromosome assigned based on the alignment of flanking sequences of markers to the *M. truncatula* genome v3.5. The first digital after “S” of the marker refers to the respective chromosome number, such as marker with“S1” refers chromosome 1, “S6” refers chromosome 6 and so on. “^∗^”, these SNPs were initially with unknown locations but reassigned to respective chromosomes by BLAST of the flanking sequence tags to the updated version of *M. truncatula* genome (Mt4.0 v1). Underlined markers with the same first 5–6 digitals are located at the same locus. The abbreviations of putative candidate genes linked to the respective significant marker loci are expanded in the legend of **Figure 5**.*

Twenty-two markers were identified by the TASSEL pipeline (**Table 4**, **Figure 5B**). Among them, 19 were located on 4 chromosomes (2, 4, 5, and 6) and the rest were of unknown location (“U” in **Table 4**). Of those with a chromosomal location, 13 were on chromosome 6, 3 on chromosome 5, 2 on chromosome 4 and one on chromosome 2. The most significant markers were S6_6801965 and S189473_1178 with *p*-values of 2.9E-9 and 4.6E-9, respectively (**Table 4**) and both markers were located on chromosome 6.

**Table 4:** Significant markers associated with VW by the TASSEL-GBS pipeline.

Trait	Marker	Allele	Chr	*P*-value	*R*^2^	Candidate
VW	Contig_195989_817	A/C	2^∗^	4.45E-06	0.15	
VW	S4_21582742	C/T	4	3.52E-07	0.15	
VW	S4_24041677	A/T	4	5.21E-08	0.20	
VW	S5_10492250	C/A	5	1.27E-07	0.19	LRR
VW	S5_10492298	A/G	5	1.27E-07	0.19	LRR
VW	S5_25672447	C/T	5	1.44E-07	0.21	PRBRP
VW	S6_6801965	A/G	6	2.92E-09	0.25	TMTP
VW	S6_30922451	C/A	6	7.88E-07	0.14	MAP
VW	S6_30922471	T/A	6	7.88E-07	0.14	MAP
VW	Contig_17722_2270	G/T	6^∗^	2.64E-08	0.21	TFIIF
VW	Contig_117822_726	T/A	6^∗^	2.06E-07	0.18	AP2
VW	Contig_149731_1411	G/T	6^∗^	1.84E-06	0.13	RBP
VW	Contig_158631_4246	G/A	6^∗^	4.59E-07	0.17	RNABP
VW	Contig_172356_2305	C/T	6^∗^	5.28E-06	0.14	
VW	Contig_173909_2230	G/A	6^∗^	7.28E-06	0.14	CAP160
VW	Contig_189473_1178	T/A	6^∗^	4.57E-09	0.21	TMP
VW	Contig_1100652_1157	T/A	6^∗^	3.44E-07	0.18	ARRF
VW	Contig_1103756_995	T/A	6^∗^	2.42E-06	0.15	VITP
VW	Contig_2313381_25389	T/C	6^∗^	1.92E-06	0.13	RCC1
VW	Contig_164981_1134	C/T	U	3.36E-08	0.18	
VW	Contig_173164_506	A/C	U	2.36E-06	0.15	
VW	Contig_2335763_95313	A/G	U	3.05E-06	0.16	

*“Chr,” chromosome assigned based on the alignment of flanking sequences of markers to the *M. truncatula* genome v3.5. The first digital after “S” of the marker refers the respective chromosome number. “U,” marker with unknown chromosome location. “^∗^,” these SNPs were initially with unknown locations but reassigned to respective chromosomes by BLAST of the flanking sequence tags to the updated version of *M. truncatula* genome (Mt4.0 v1). Underlined markers with the same first 6 digitals are located at the same locus. The full names of the abbreviations of putative candidate genes linked to the respective significant marker loci are: MAP, MAP kinase-like protein; AP2, AP2 domain class transcription factor; RBP, double-stranded RNA-binding motif protein; RNABP, RBD nucleic acid binding protein; RCC1, chromosome condensation regulator RCC1 repeat protein. The rest abbreviations are expanded in the legend of **Figure 5**.*

Twenty-one markers were identified by the UNEAK pipeline (**Table 5**, **Figure 5C**). Among them, 16 were located on 4 chromosomes. The location of the reminding 5 markers was unknown (“U” in **Table 3**).

**Table 5:** Significant markers associated with VW by the UNEAK pipeline.

Trait	Marker	Allele	Chr	*P*-value	*R*^2^	Candidate
VW	S3_151767048	G/T	3	6.97E-06	0.15	TMP
VW	S5_114680024	C/A	5	4.84E-06	0.15	PPR
VW	S5_59656021	G/A	5	9.28E-06	0.13	PRBRP
VW	S5_59951052	A/T	5	1.77E-09	0.25	NAATP
VW	S6_162766039	T/C	6	2.12E-08	0.22	TMTP
VW	S6_31221028	C/A	6	5.01E-08	0.22	VITP
VW	S6_96132030	A/T	6	9.50E-08	0.20	
VW	S6_39177057	A/G	6	3.03E-07	0.21	DUF1442
VW	S6_36346047	C/T	6	3.05E-07	0.20	
VW	S6_106882043	C/A	6	5.87E-06	0.14	
VW	S6_122257050	T/A	6	6.17E-06	0.17	ARRF
VW	S6_6332023	C/A	6	9.44E-06	0.13	
VW	S6_39031030	C/A	6	9.88E-06	0.15	
VW	S6_157111050	A/G	6	9.95E-06	0.15	Tic22
VW	S7_15550032	G/T	7	2.20E-06	0.17	
VW	S7_131660043	C/G	7	4.44E-06	0.15	ABC_TP
VW	S0_11349018	T/G	u	1.02E-07	0.23	
VW	S0_126618054	C/T	u	6.56E-07	0.17	45S
VW	S0_32496031	A/C	u	2.36E-06	0.16	TPS
VW	S0_101807021	G/T	u	4.00E-06	0.18	
VW	S0_18200045	A/G	u	8.55E-06	0.14	

*“Chr,” chromosome assigned based on the alignment of flanking sequences of markers to the *M. truncatula* genome v3.5. The first digital after “S” of the marker refers the respective chromosome number. “S0” or “U” refers unknown chromosome location. Underlined markers with the same first 6 digitals are located at the same locus. The full names of the abbreviations of putative candidate genes linked to the respective significant marker loci are: PPR, pentatricopeptide repeat protein; DUF1442, DUF1442 family protein; 45S, 45S ribosomal RNA intergenic spacer; TPS, trehalose-6-phosphate synthase. The rest abbreviations are expanded in the legend of **Figure 5**.*

Of those with known location, 10 markers were on chromosome 6, 3 on chromosome 5, 2 on chromosome 7 and one on chromosome 3 (**Table 5**). The most significant markers were S5_59951052 (*p* = 1.77E-9), S6_162766039 (*p* = 2.12E-8), S6_31221028 (*p* = 5.01E-8) and S6_96132030 (*p* = 9.50E-8). The former was located on chromosome 5 and the later three were on chromosome 6 (**Table 5**).

### Assigning the Loci Associated With VW Resistance to Known Genes

To identify potential candidate genes linked to marker loci associated with VW resistance, a BLAST search was performed as described in Section “Materials and Methods.” Of significant markers identified by the FreeBayes pipeline, 10 linked to 5 known genes in the *M. truncatula* genome (**Table 3**). Among them, three markers (AC146807-58-93588, -93595 and -93604) located at the same locus on chromosome 1 linked to the same gene, E3 ubiquitin-protein ligase (E3_Ubl). Two markers (S6_10615509 and S6_10615526) at the same locus on chromosome 6 linked to the DNA primase (DNA_P), an enzyme involved in the replication of DNA. On the same chromosome, markers contig_100652_1157 and contig_59661_3280 linked to the ankyrin repeat RF-like protein (ARRF) and Tic22 family protein (Tic22), respectively. Three additional markers (Contig_73909_2401, _2417 and _2437) at the same locus linked to the CAP160 repeat protein (CAP160).

Of significant markers identified by the TASSEL pipeline (**Table 4**), two markers (S5_10492250 and S5_10492298) at the same locus on chromosome 5 linked to a leucine-rich repeat receptor kinase (LRR). On the same chromosome, marker S5_25672447 linked to a pumilio-family RNA-binding repeat protein (PRBRP). A number of known genes were linked to significant markers on chromosome 6. A transmembrane amino acid transporter protein (TMTP) linked to marker S6_6801965. A MAP kinase (MAP) was linked to two markers (S6_30922451 and S6_39022471) located at the same locus. Additional markers (**Table 4**, “6^∗^”) were initially with unknown chromosome position but reassigned to chromosome 6 by BLAST search using their flanking sequences against the updated version of *M. truncatula* genome (Mt4.0 v1), including Contig_17722_2270 linked to the transcription factor IIF beta subunit (TFIIF), Contig_117822_726 linked to AP2 transcription factor (AP2), Contig_189473_1178 linked to a transmembrane protein (TMP), Contig_158631_4246 linked to a RNA-binding protein (RNABP), Contig_110756_995 linked to the vacuolar iron transporter-like protein (VITP), and Contig_2313381_25389 linked to the chromosome condensation regulator RCC1 repeat protein (RCC1). Interestingly, two genes, ARRF and CAP160 linked to Contig_1100652_1157 and Contig_173909_2230 respectively, were also identified by the FreeByes pipeline.

Among significant markers identified by the UNEAK pipeline (**Table 5**), five were linked to the same genes (TMP, PRBRP, TMTP, VITP and ARRF) identified by the TASSEL, and two (Tic22 and ARRF) identified by the FreeByes pipelines. Additional markers were only identified by the UNEAK pipeline, including S5_114680024 and S5_59951052 on chromosome 5 linked to pentatricopeptide repeat (PPR) and neutral amino acid transporter proteins (NAATP), respectively, S6_19276039 on chromosome 6 linked to transmembrane amino acid transporter protein (TMAATP), and S6_96132030 and S6_39177057 linked to unusual kinase (UK) and DUF1442 family protein (DUF1442), respectively. Interestingly, a disease resistance gene, drug resistance transporter-like ABC domain protein (ABC_TP) was linked to S7_131660043 on chromosome 7. Among markers with unknown chromosomal position, two markers, S0_126618054 and S0_32496031 were linked to 45S ribosomal RNA intergenic spacer (45S) and trehalose-6-phosphate synthase (TPS), respectively.

## Discussion

### Comparison of GBS Pipelines Used for Genotype Calling

Among the three pipelines used for genotype calling in the present study, TASSEL and UNEAK were based on single SNPs while the FreeBayes was a haplotype-based pipeline. Both TASSEL and FreeBayes used *M. truncatula* as a reference genome for sequence alignment, whereas the UNEAK used a network approach without a reference genome for SNP discovery. Our results are consistent with other reports showing that different SNP callers identify different SNPs from the same input files using the same parameters (O’Rawe et al., 2013; Yu and Sun, 2013; Li, 2014; Clevenger et al., 2015). Therefore, the choice of SNP calling software is crucial when developing a SNP calling pipeline. For instance, in the present study, the FreeBayes pipeline may be more appropriate for genotype calling in autotetraploid alfalfa as it can handle haplotype-based variants including single and multiple nucleotide polymorphisms, allelic series of tri-SNPs and tetra-SNPs, MNPs, and indels with a variable number of nucleotides. We used the FreeBayes pipeline for genotyping in a diverse panel of accessions of alfalfa and identified a number of haplotype loci for drought resistance traits (Zhang et al., 2015) and in a breeding population for VW resistance (Yu et al., 2016). In the present study, compared to the TASSEL and UNEAK pipelines, although a lower missing value was found in FreeBayes, fewer variants were obtained (**Table 2**) and only13 markers significantly associated with VW resistance were identified by the FreeBayes pipeline (**Table 3**). The TASSEL pipeline generated the most variants and identified 22 significant SNP markers associated with VW resistance (**Table 4**). The number of significant markers (21 SNPs) identified by the UNEAK was comparable (**Table 5**) to that of the TASSEL pipeline. This may due to the relatively higher coverages were obtained by TASSEL (11.7X) and UNEAK (5.2X) than that by the FreeBayes pipeline (1.4X) (**Table 3**). Nevertheless, each pipeline identified major resistance loci to VW on chromosome 6, suggesting a consistency of effectiveness among the three pipelines used in the present study in the identification of VW resistance.

### Putative Candidate Genes Linked to Marker Loci for VW Resistance

The whole genome sequence of *M. truncatula* provides a useful database for searching for candidate genes underlying the marker loci associated with VW resistance in alfalfa as they are close relatives. Our BLAST search results showed that 29 functional genes are linked to 36 markers identified by the three pipelines in the present study. Most of significant markers were located on chromosome 6 where shared linked genes were identified by at least two pipelines used in the present study. One of them was the ankyrin repeat protein, ARRF that was identified by all pipelines (Supplementary Table S4). The ankyrin repeat protein is a specific protein associated with the plasma membrane and provides for the interaction of the cytoskeleton with integral membrane proteins. It has been reported that the *Arabidopsis* ankyrin repeat protein, NPR1, provides for the onset of systemic acquired resistance (SAR) to a broad spectrum of pathogens. Defective mutants fail to respond to SAR-inducing treatments, inhibiting the expression of pathogenesis-related (PR) genes and exhibiting increased susceptibility to pathogen infections (Cao et al., 1997).

Among the resistance loci identified in the present study, two markers (S5_10492250 and S5_10492298) with the same location (48 bases away) on chromosome 5 were linked to a member of the TIR-NBS-LRR gene family. The NBS-LRR gene family has been well-documented and can be divided into two subfamilies, TIR-NBS-LRR and non-TIR-NBS-LRR (DeYoung and Innes, 2006; Grau et al., 2006; McHale et al., 2006). The plant NBS-LRR gene family contains a large class of disease resistance genes, known as R genes. The TIR-NBS-LRR genes play roles in disease resistance in plants. There is evidence to suggest that the TIR-NBS-LRR gene (RCT1) from *M. truncatula* confers resistance to anthracnose in transgenic alfalfa plants (Yang et al., 2008). The identification of markers linked to the TIR-NBS-LRR gene in the present study suggests their contribution to VW resistance.

We identified an ABC transporter linked to the significant marker, S7_131660043 on chromosome 7 in the present analysis. The ABC transporters are known to participate in many processes including polar auxin transport, alkaloid import, tissue pigmentation, vacuolar xenobiotic sequestration, stomatal regulation, disease resistance, lipid catabolism, antibiotic resistance, assembly of redox-active cytosolic Fe/S proteins, and heavy metal tolerance (Rea, 2007). One of the mechanisms of plant-pathogen reaction is the reduction of accumulation of toxic compounds at the target site due to secretion by ABC transporters that can transport a wide variety of natural products, including plant antimicrobials (Stergiopoulos et al., 2002; Roohparvar et al., 2007). Tic22 is a chaperone that is essential for protein translocation into the apoplast (Glaser et al., 2012). VITP is involved in the transfer of iron from the cytosol to the vacuole for intracellular iron storage (Kim et al., 2006). CAP160 was identified as a cold and desiccation responsive protein. CAP160’s function may be involved in participating in the assembly of ribosomes or to help accelerate translation (Kaye et al., 1998). Interestingly, one of the significant marker loci identified by the UNEAK pipeline linked to a trehalose-6-phosphate synthase, the first enzyme of the trehalose synthesis pathway. It has been reported that trehalose accumulation in rice plants confers high tolerance levels to different abiotic stresses (Garg et al., 2002). It has been also suggested that trehalose-6-phosphate synthase regulates primary and secondary metabolism during infection by the rice blast fungus (Fernandez and Wilson, 2011).

### Polygenic Inheritance of VW Resistance in Alfalfa

The genetic basis of alfalfa resistance to VW has been investigated by several research groups. Viands (1985) suggested that complex additive effects contribute to VW resistance in alfalfa cultivar ‘Vertus’. Miller and Christie (1991) reported that general combining ability (GCA) significantly influences the expression of resistance to VW in ‘Vertus’ alfalfa and suggested that additive genetic variance is the primary source of VW resistance. Our previous reports (Zhang et al., 2014; Yu et al., 2016) identified multiple loci associated with VW resistance in two alfalfa mapping populations. This assumption is supported by the present study where multiple loci are shown to contribute to VW resistance in F1 population of alfalfa segregated for VW resistance.

QTLs for VW resistance have been reported in *M. truncatula*. They include a major QTL on chromosome 7, and two minor QTLs on chromosomes 2 and 6 (Ben et al., 2013). In our previous study, three resistance loci associated with VW resistance were identified on chromosome 7 and one on chromosome 2 (Zhang et al., 2014). Our recent report identified multiple loci associated with VW resistance on chromosomes 5, 6, 7, and 8 (Yu et al., 2016). In the present study, most significant resistance loci have been identified at similar locations on chromosomes 5, 6, and 7, suggesting common pathways for VW resistance in *M. sativa* and *M. truncatula*. However, additional resistance loci to VW have been identified in *M. sativa* by both our previous (Zhang et al., 2014; Yu et al., 2016) and the present studies, whereas these loci were not found in *M. truncatula*. For instance, several VW resistance loci were identified on chromosomes 5 and 8 by our previous (Zhang et al., 2014; Yu et al., 2016) and the present studies, whereas no VW resistance locus has been reported on chromosomes 5 and 8 in *M. truncatula.*

## Conclusion

We used three GBS pipelines for SNP discovery and identification of marker loci associated with VW resistance in the F1 alfalfa population using genotyping-by-sequencing and genome-wide association approaches. This is the first effort to compare the effect of pipelines on SNP discovery in alfalfa. Although different markers were identified by different pipelines, most significant resistance loci located on chromosome 6 have been identified by all pipelines, confirming the consistency of SNP discovery and marker identification by GBS pipelines used in the present study. Sequence alignment to the *M. truncatula* genome revealed multiple resistance loci, several of which are linked to known resistance genes. The putative resistance loci thus identified had similar chromosomal locations to those previously reported in tetraploid alfalfa (Zhang et al., 2014; Yu et al., 2016) and in its diploid relative, *M. truncatula* (Ben et al., 2013). With further validation, those markers closely linked to VW resistance can be used for MAS to accelerate the development of new alfalfa cultivars with improved resistance to VW.

## Author Contributions

Conceived and designed the experiments: L-XY. Performed the experiments: SB, X-PL. Analyzed the data: PZ and DM. Wrote the paper: L-XY and PZ.

## Conflict of Interest Statement

The authors declare that the research was conducted in the absence of any commercial or financial relationships that could be construed as a potential conflict of interest.

## Footnotes

1 https://www.naaic.org/

2 http://www.ibm.com/software/analytics/spss/

3 http://picard.sourceforge.net/

4 http://ape-package.ird.fr/pegas.html

5 http://www.maizegenetics.net/\#!tassel/c17q9

6 http://www.ncbi.nlm.nih.gov

7 http://phytozome.jgi.doe.gov/jbrowse/index.html?data=genomes\%2FMtruncatula\&loc

8 http://www.phytozome.net/
